# Professional Notes

**Published:** 1887-11-12

**Authors:** Geo. W. Potter


### Professional Notes.

By Geo. W. Potter, M.D.

There are still to be found among medical men some who do not believe in what is called the "antiseptic treatment." Among intelligent practitioners opinions may differ about the details of Listerism, but there can be no doubt at all as to the prime advantages of absolute cleanliness in surgery; and cleanliness is really the most perfect antisepticism. On this point Sir William Stokes, in his recent address at the opening of the session of the College of Surgeons in Ireland, gave some statistics which are worthy of the most serious consideration on the part of those who may be still in doubt. He said: "As a proof of what we have gained in our power of warding off and disarming what are not inaptly termed 'preventible' diseases following wounds, all more or less connected with septic infection, let us briefly glance at some statistics connected with comparatively recent wars, for which I am indebted to my friend, Sir William MacCormac. In the Crimean War the number of men who lost their lives in the French army was 95,615, of whom only 10,240 perished at the hands of the enemy, the remainder succumbing to diseases resulting directly from their wounds. In the American war 95,000 men died from wounds, whilst 184,000, nearly double the number, perished from septic affections consequent on them. A remarkable change for the better was observed in the Franco-German war. In this campaign 28,282 died of their wounds, while only 12,253 died of preventible disease. But the greatest result of all yet obtained was in the Egyptian campaign of 1882, in which not a single instance of death from pyæmia, septicæmia, erysipelas, or hospital gangrene is recorded."

On the question of "change in the methods of practice" Sir William Stokes, addressing students, says: "In your professional life hereafter, if slow to accept new principles and practice, be also slow to reject them. Remember there is nothing so cheap or so nasty as senseless detraction; nothing so vulgar, so stupid and contemptible as an unreasoning scepticism; and that indulgence in either or both has not infrequently brought persons into a position at once melancholy, and at the same time ludicrous. I allude more particularly to the hostility that was formerly evinced by 'practical' persons to the many instruments of precision now so indispensable to every well-educated practitioner, and of late years, mainly, I regret to say, in the surgical ranks of our profession, to the principles and practices of antisepticism as elaborated by Pasteur and Lister. When we consider how recently we have heard antiseptic practices held up to contempt as little else than a fashionable, but essentially ephemeral craze, and its professors and advocates described as persons apparently temporarily afflicted with a form of harmless lunacy, which in time, and with a more extended experience, they may reasonably be expected to recover from—we have a good example of the humiliating position unthinking detractors may eventually find themselves in."

At a recent meeting of the Clinical Society of London several cases of suppurative peritonitis were reported which had been treated by opening the abdomen, washing and sponging the peritoneum. Mr. Richard Barwell read notes of a case in which "it being evident that the man had a bad type of acute peritonitis," the abdomen was opened "in the middle line below the umbilicus. A large quantity of gas, not of fæculent odour, escaped. No rupture of any viscus was found, but in its lower part the peritoneum contained a quantity of thick pus; there were no adhesions; parts of the intestines were congested, and the membrane was somewhat thickened. Three sponges passed into the lowest part of the cavity were withdrawn covered with tenacious flocculent pus. A smooth-nozzled glass funnel was then deeply introduced, and the lower part of the cavity washed out with ten pints of distilled water, at a temperature of 99 deg., bringing away quantities of pus and flocculi. After sponging, a second and smaller washing and sponging was directed to the upper part. The abdomen was then sewn close without any drain. The whole operation lasted an hour." The man convalesced rapidly, and made a good recovery. This case, commencing with a blow, well illustrates the value of intelligence and courage in practice. Not many years ago such a patient would inevitably have died for want of these two qualities. From the previous history and the symptoms, Mr. Barwell judged that the peritonitis had progressed to suppuration. The state of matters then might have been stated thus: Leave the pus, and the patient will die; remove the pus and the patient may recover. It was clearly, therefore, the duty of the surgeon to operate; as it would have been the duty of a general practitioner in similar circumstances if no operating surgeon had been at hand. But how few general practitioners would have operated! We call attention to the case as one of a kind which may at any time occur in a remote country district, and in which the family attendant, after fully explaining the possibilities to the friends, should operate without hesitation. There is no doubt whatever that many lives are lost annually in outlying districts on account of the cowardice of the practitioner—a cowardice fostered by many teachers during the student's career.

"Cremation" formed the subject of an address given by Sir Spencer Wells at the inaugural meeting of the Nottingham Medico-Chirurgical Society. The public sentiment is gradually changing, and many persons of influence in the religious world are inclining towards the use of this method of burial on sanitary grounds. The medico-legal objection that a murdered or poisoned body might be burned, and that discovery of crime by subsequent exhumation would then be impossible, was answered by the argument that under proper regulations no human body could possibly be burned without far more positive evidence of death from natural or known causes than was now required before burial, and that by promoting cremation they were also leading to a more correct and general system of registration and certification of the true cause of death. The religious objection was met by the words of the late Bishop of Manchester and Canon Liddon. The strongest objection was the sentimental one—the fear of offending relatives and friends, and the disinclination at a time of bereavement and suffering to depart from ordinary routine or custom. Sir Spencer Wells firmly believed from his own experience that, however painful it might be to make people understand what a revolting change takes place after burial in the earth, and how dangerous it was to the living, very little more was required to call forth a purer sentiment in favour of rapid purification by fire. Cremation is sometimes objected to on the score of expense; but Sir Spencer stated that if the practice became general, the cost to a parish or union need not exceed ten shillings for each cremation, and that from ten to twenty pounds would cover the expenditure of each funeral of persons of the middle and upper classes.

The battle of the microbes is still going forward, and it is necessary for every medical man of critical judgment to keep himself abreast of the state of knowledge and opinion. The *British Medical Journal* publishes a note by M. E. Metschnikoff on the *Struggle of the Cells of the Organism against invading Microbes*. Certain cells normal to the organism appear to have the power of absorbing and digesting microbes. These cells Metschnikoff calls "phagocytes." The action of the phagocytes is not confined to the absorption of dead or degenerated material; they also furnish the organism with a means of resisting microbes which may have penetrated into the tissues. The experimenter made his researches on *transparent* animals, such as daphnea, which are often invaded by parasites of the yeast family. The spores of this parasite, in the form of long needles, penetrate with the food into the intestine, through which they escape into the body-cavity of the daphnea. As soon as they have done so, a struggle commences between them and the white corpuscles, which, isolated or in groups, absorb the spore and destroy it, transforming it into shapeless granules. If the white corpuscles are successful the daphnea is saved. But sometimes—and here is the point of interest in the conflict—the spores escape the white corpuscles, and then they germinate, and a considerable number of the conidia invade the body of the animal and kill it. Similarly it is stated that in man and the other higher vertebrates there is a struggle between the microbes and the cell elements, but the phenomena are of course more complicated. There are in man two kinds of "phagocytes," the leucocytes and the connective-tissue corpuscles. The former Metschnikoff calls "microphagi," and the latter "macrophagi." Among the macrophagi he also includes the epithelial cells of the pulmonary alveoli, and in general all those which are provided with only one large nucleus, and which are capable of absorbing solid bodies. In these conclusions there is nothing contradictory of well-known facts in the history and development of the lower forms of life in the vegetable and animal kingdoms; and a continuance of research on the same lines may be expected to make clear and simple many circumstances which now seem to be enshrouded in mystery.

